# Comparison of Three Quality of Life Instruments in Lymphatic Filariasis: DLQI, WHODAS 2.0, and LFSQQ

**DOI:** 10.1371/journal.pntd.0002716

**Published:** 2014-02-20

**Authors:** Cristina Thomas, Saravu R. Narahari, Kuthaje S. Bose, Kuthaje Vivekananda, Steven Nwe, Dennis P. West, Mary Kwasny, Roopal V. Kundu

**Affiliations:** 1 Department of Dermatology, Northwestern University Feinberg School of Medicine, Chicago, Illinois, United States of America; 2 Department of Integrative Dermatology, Institute of Applied Dermatology, Uliyathadka, Kasaragod, Kerala, India; 3 Department of Preventative Medicine Biostatistics Collaboration Center, Northwestern University Feinberg School of Medicine, Chicago, Illinois, United States of America; University Clinic Bonn, Germany

## Abstract

**Background:**

The Global Program to Eliminate Lymphatic Filariasis aims to interrupt transmission of lymphatic filariasis and manage morbidity in people currently living with the disease. A component of morbidity management is improving health-related quality of life (HRQoL) in patients. Measurement of HRQoL in current management programs is varied because of the lack of a standard HRQoL tool for use in the lymphatic filariasis population.

**Methodology/Principal Findings:**

In this study, the psychometric properties of three health status measures were compared when used in a group of lymphatic filariasis patients and healthy controls. The World Health Organization Disability Assessment Schedule 2.0 (WHODAS 2.0), the Dermatology Life Quality Index (DLQI), and the Lymphatic Filariasis Quality of Life Questionnaire (LFSQQ) were administered to 36 stage II and stage III lymphatic filariasis subjects and 36 age and sex matched controls in Kerala, India. All three tools yielded missing value rates lower than 10%, suggesting high feasibility. Highest internal consistency was seen in the LFSQQ (α = 0.97). Discriminant validity analysis demonstrated that HRQoL was significantly lower in the LF group than in controls for the WHODAS 2.0, DLQI, and LFSQQ, but total HRQoL scores did not differ between stage II and stage III lymphedema subjects. The LFSQQ total score correlated most strongly with the WHODAS 2.0 (r = 0.91, p<0.001) and DLQI (r = 0.81, p<0.001).

**Conclusions/Significance:**

The WHODAS 2.0, DLQI, and LFSQQ demonstrate acceptable feasibility, internal consistency, discriminate validity, and construct validity. Based on our psychometric analyses, the LFSQQ performs the best and is recommended for use in the lymphatic filariasis population.

## Author Summary

Lymphatic filariasis affects approximately 120 million people and is the second leading cause of life-long disability worldwide. Because lymphatic filariasis is one of the World Health Organization's six eradicable diseases, much effort has been placed into reducing transmission of the disease and managing morbidity. Novel interventions frequently use health-related quality of life as an outcome measure to monitor efficacy of the intervention. In an effort to delineate the strengths and weaknesses of health status measures and recommend use of a single tool in the lymphatic filariasis population, we compared the use of three health status tools (The World Health Organization Disability Assessment Schedule 2.0, the Dermatology Life Quality Index, and the Lymphatic Filariasis Quality of Life Questionnaire) in lymphatic filariasis subjects and healthy controls in Kerala, India. The Lymphatic Filariasis Quality of Life Questionnaire performed the best by discriminating well between subjects and controls, possessing significant content overlap with the other two tools, yielding a low missing value rate, and being internally consistent. This is the first study to compare health status measures in lymphatic filariasis subjects and provides insight into the use of the tools in quality of life analysis.

## Introduction

Lymphatic filariasis (LF) is the second leading cause of disability worldwide, affecting almost 120 million people across 81 countries [1]. Although LF is not curable, the Global Program to Eliminate Lymphatic Filariasis has reduced disease transmission and is working to expand morbidity management programs globally [2], [3]. Morbidity management is expected to greatly improve health-related quality of life (HRQoL) in LF patients because LF is characterized by disfiguring lymphedema and debilitating inflammatory episodes that cause significant disability and social isolation [4]–[6]. In fact, HRQoL and health status assessments have become primary measures of intervention efficacy in LF studies [7]–[11].

Despite the reliance on HRQoL in LF management, no consensus has been made on the most suitable tool for use in the LF population. Generic instruments, including a seven domains and five levels (7D5L) tool, the International Classification of Functioning, Disability and Health checklist, and the World Health Organization Disability Assessment Schedule 2.0 (WHODAS 2.0), have been used in the LF population because of their applicability to a number of diseases and the ease of HRQoL comparison among diseases [8], [12]–[14]. However, most generic instruments are only able to measure 50% of LF-associated problems, often overlooking feelings of fear and embarrassment [15]. Emphasis has now shifted to disease-specific tools that are better able to measure disease-specific factors as well as disease progression [15], [16]. Despite the call for development of LF-specific tools, only two such tools exist, the LF QoL Tool (LF-QoL) and the LF-specific QoL Questionnaire (LFSQQ) [7], [10], [17], [18]. Of the two tools, the LFSQQ has been used to measure intervention efficacy and has been tested in a larger patient population. Because generic measures miss relevant HRQoL content and disease-specific tools have only recently been developed, LF HRQoL has been most often measured by the Dermatology Life Quality Index, a skin-specific HRQoL tool [11], [19]–[21]. However, the DLQI has been found to assess only 24% of disability caused by LF [15].

Given the lack of a standard HRQoL tool for LF and minimal research comparing the various instruments, the aim of this study was to compare the relative performances of three health status measures in LF subjects and a matched control group. The tools chosen included a generic tool, the WHODAS 2.0, a skin-specific tool, the DLQI, and a disease-specific tool, the LFSQQ. Specifically, we examined four instrument psychometric properties: feasibility, reliability, discriminative validity, and construct validity.

## Methods

### Ethics Statement

Ethical clearance was obtained from the institutional review boards at Northwestern University in Evanston, Illinois USA, the Institute of Applied Dermatology in Kasaragod, Kerala India, and the Speciality Hospital in Kannur, Kerala, India. Within the LF population in Kerala, written consent was unable to be obtained because subjects did not want their name associated with the diagnosis of LF due to the disease's cultural stigma. Because of this limitation, verbal consent was obtained from all participants. As detailed in the IRB-approved protocol, the consent document was read aloud to participants and signed by the reader upon participant's approval to signify the participant's consent.

### Study Population

In this cross-sectional study, a total of 36 LF subjects were consecutively enrolled from the outpatient units of the Institute of Applied Dermatology in Kasaragod, Kerala, India and the Speciality Hospital in Kannur, Kerala, India. Subjects were enrolled if they had a clinical diagnosis of LF, had never been treated for LF, and were at least 18 years of age. LF staging was performed by physicians based on the International Society of Lymphology's consensus staging system (Table 1) [22]. Thirty-six control subjects were age and sex matched to LF subjects and were recruited from Kasaragod, Kerala. Control inclusion criteria were: no history of LF diagnosis, no blood relation to cases included in the study, and at least 18 years of age.

**Table 1:** International Society of Lymphology consensus staging system.

Stage	Criteria
I	Fluid accumulation that subsides with limb elevation. Pitting edema may be present.
II	Fluid accumulation that does not subside with limb elevation. Pitting edema may be present.
III	Fluid accumulation that does not subside with limb elevation and presence of trophic skin changes (hyperpigmentation, fat deposition, or warts)

### Design and Instruments

All subjects completed the WHODAS 2.0, DLQI, and LFSQQ in the local language of Malayalam. Because the WHODAS 2.0 was not available in Malayalam, the original English version was translated according to standard protocols [23]. Two native Malayalam speakers independently forward-translated the WHODAS 2.0 from English to Malayalam. Both translators and the local team assessed the tool's clarity, cultural relevance, and language, and any differing opinions were discussed. Changes to the instrument were made as needed. This version was then back-translated to English and confirmed to be equivalent with the original English version to ensure the validity of the Malayalam version. The DLQI and LFSQQ were available in Malayalam and have been validated for use [7], [24]. The sequence of instrument administration was randomized to avoid an ordering bias. Demographic information was obtained via a demographic questionnaire. Table 2 lists the domains of each tool and outlines tool domains that cover similar content.

**Table 2:** Content comparison of WHODAS 2.0, DLQI, and LFSQQ.

WHODAS 2.0	DLQI	LFSQQ
Cognition	–	Psychological Health
Mobility	–	Mobility
–	Symptoms & Feelings	Pain/Discomfort Disease Burden
Getting Along Participation	Personal Relationships	Social Participation
Self-Care	–	Self-Care
Life Activities	Leisure Work/School Daily Activities	Usual Activities
–	Treatment	–

The WHODAS 2.0 is a generic health and disability assessment tool that describes effects of disease on six domains: cognition, mobility, self-care, getting along, life activities, and participation in society [25]. Disability perception is measured by responses on a 5-point scale from 1 (no difficulty) to 5 (extreme difficulty or cannot do). Final scores were calculated using a WHO SPSS 36 version syntax for employed subjects and a WHO SPSS 32 version syntax for unemployed subjects. The WHO SPSS 32 version syntax is identical to the WHO SPSS 36 version syntax with the omission of four questions relating to work ability. Domain and total scores range from 0 to 100 with a higher score indicating greater impairment of health status.

The DLQI is a 10-item questionnaire that assesses skin-specific HRQoL through six domains: individual's symptoms and feelings, daily activities, leisure, work and school, personal relationships, and treatment [26]. Questions are scored on a 4-point scale, resulting in a maximum score of 30, or large negative effect on HRQoL, and a minimum score of 0, or no effect on HRQoL.

The LFSQQ was developed to assess quality of life in LF subjects through seven domains: mobility, self-care, usual activities, disease burden, pain/discomfort, psychological health, and social participation. Each item is scored on a 5-point scale (no problem, mild, moderate, severe, most severe). Total scores are calculated based on the number of questions answered and the raw scores [7]. Scores range from 0 to 100 with a higher score indicating a higher HRQoL.

### Statistical Analyses

Median scores and interquartile ranges for the WHODAS 2.0, DLQI, and LFSQQ were calculated for subject and control data. Discriminant ability, or the ability of a questionnaire to discriminate between respondent subgroups, was gauged by comparing HRQoL scores between subjects and controls using Wilcoxon Rank Sum tests. Further comparisons were made adjusting for demographic and disease-specific variables using linear regression models.

A number of factors account for the overall feasibility of any tool, including cost, completion time, and ease of administration. In this study, feasibility was evaluated by examining the number of missing item responses. Internal consistency is a measure of the consistency of results yielded by items of a single construct. Cronbach's α-coefficient was calculated to determine the internal consistency of the WHODAS 2.0 and the LFSQQ. Coefficient values ≥0.70 were deemed reliable [27]. Internal consistency values were not calculated for the DLQI domains because domains consisted of only one or two items. A test demonstrates construct validity if it accurately measures the construct it intends to measure. Construct validity was assessed by comparing correlations between related constructs of the three tools using Spearman's rank correlation coefficients. Correlations were regarded as weak if the Spearman's coefficient was less than 0.50, moderate if the coefficient was between 0.50 and 0.69, and strong if the coefficient was greater than 0.69.

## Results

Thirty-six subjects and 36 controls participated in this study. Demographic information for LF subjects and healthy controls are summarized in Table 3. For both groups, more females participated than males. LF subjects were more likely to have no education (36.1%) than controls in which no participants lacked education. All other variables were comparable between LF subjects and the control group including mean age (51.7 vs. 52.3 years), primary source of family income (16.7% vs. 25% as primary source), and poverty level (25% vs. 44.4% below poverty line). Disease-specific variables of subjects are shown in Table 4. Of the subjects, 58.3% had stage II lymphedema and 41.7% had stage III lymphedema.

**Table 3:** Study subjects' demographic data.

Characteristic	LF Subjects (n = 36)	Controls (n = 36)
No. of females	28 (77.8%)	28 (77.8%)
Age in years (mean±SD)	51.7±16.2	52.3±16.1
Education*		
No education	13 (36.1%)	0 (0.0%)
<5 years completed	7 (19.4%)	4 (11.1%)
5–9 years completed	5 (13.9%)	5 (13.9%)
10–12 years completed	7 (19.4%)	18 (50.0%)
College completed	4 (11.1%)	9 (25.0%)
Marital Status		
Unmarried	6 (16.7%)	8 (22.2%)
Married/engaged	24 (66.7%)	23 (63.9%)
Divorced	1 (2.8%)	0 (0.0%)
Widowed	5 (13.9%)	5 (13.9%)
Occupation		
Full-time	9 (25.0%)	18 (50.0%)
Part-time	2 (5.6%)	0 (0.0%)
Unemployed	1 (2.8%)	0 (0.0%)
Housewife	23 (63.9%)	2 (5.6%)
Retired	1 (2.8%)	16 (44.4%)
Primary source of family income	6 (16.7%)	9 (25.0%)
Poverty level		
Above poverty line	27 (75.0%)	20 (55.6%)
Below poverty line	9 (25.0%)	16 (44.4%)
Source of drinking water		
Tap	11 (30.6%)	7 (19.4%)
Well	24 (66.7%)	28 (77.8%)
Pond, lake, or body of water	1 (2.8%)	1 (2.8%)
History of hypertension	10 (27.8%)	7 (19.4%)
History of diabetes	5 (13.9%)	5 (13.9%)

* Significant difference between groups.

**Table 4:** LF subjects' disease characteristics.

Characteristic	LF Subjects (n = 36)
Disease stage at recruitment	
Stage II	21 (58.3%)
Stage III	15 (41.7%)
Disease activity	
Progressive	12 (33.3%)
Stable	19 (52.8%)
Improving	5 (13.9%)
No. of limbs affected	
One limb	31 (86.1%)
Two limbs	5 (13.9%)
Wounds present on affected limb or limbs	8 (22.2%)
Age in years at onset of disease (mean±SD)	33.3±17.9
Duration of disease in years (mean±SD)	17.8±14.8
No. of ADLA attacks annually (mean±SD)	3.6±4.8
Family history of disease	8 (22.2%)

### Feasibility

Missing values of tools are shown in Table 5. Individual domains of the WHODAS 2.0 and DLQI showed low missing value rates (WHODAS 2.0: ≤0.1%, DLQI: 0.0%). Missing values for the LFSQQ domains were <7%, except for the usual activities domain (32.9%). Less than 10% of data for total scores of all instruments were missing (WHODAS 2.0 = 0.04%, DLQI = 0.0%, LFSQQ = 7.07%).

**Table 5:** Missing values and internal consistency.

Questionnaire	N (%) missing	Cronbach's α
WHODAS 2.0:	1 (0.04%)	0.93
Cognition (6)	0	0.85
Mobility (5)	0	0.91
Self-Care (4)	0	0.78
Getting Along (5)	0	0.85
Life Activities (8)	0	0.88
Participation in Society (8)	1 (0.1%)	0.82
DLQI:	0	0.73
Symptoms & Feelings (2)	0	
Daily Activities (2)	0	
Leisure (2)	0	
Work & School (1)	0	
Personal Relationships (2)	0	
Treatment (1)	0	
LFSQQ:	224 (7.07%)	0.97
Mobility (8)	1 (0.1%)	0.88
Self-Care (5)	25 (6.9%)	0.74
Usual Activities (7)	166 (32.9%)	0.80
Disease Burden (5)	11 (3.1%)	0.71
Pain/Discomfort (7)	20 (4.0%)	0.69
Psychological Health (7)	1 (0.2%)	0.89
Social Participation (5)	0	0.90

Number in parentheses indicates number of items.

### Reliability

Cronbach's alpha coefficients are presented for the three tools in Table 5. Whole instrument reliability was highest for the LFSQQ (α = 0.97) as compared to the WHODAS 2.0 (α = 0.93) and DLQI (α = 0.73). Domains of the WHODAS 2.0 demonstrated higher internal consistency (mean α = 0.85; range = 0.76–0.91) than the domains of the LFSQQ (mean α = 0.80; range = 0.69–0.90). All domains of the WHODAS 2.0 and all but one domain of the LFSQQ (pain/discomfort) showed internal consistency above 0.70, the minimum value of acceptable internal consistency.

### Discriminant Validity

Descriptive statistics for each tool in LF subjects and the control group are summarized in Table 6. All three tools demonstrated lower HRQoL in LF subjects as compared to the control group. The LFSQQ domains discriminated best between the two groups as all but one of the domains (self-care) yielded p values <0.001. For all three tools, domains relating to mobility, symptoms, and participation in society yielded notable differences between subject and control scores.

**Table 6:** WHODAS 2.0, DLQI, and LFSQQ score distribution.

Questionnaire	Patients	Controls	*P*
WHODAS 2.0 Domains:			
Cognition	15 (3–20)	0 (0–3)	<0.001
Mobility	50 (19–69)	0 (0–13)	<0.001
Self-Care	5 (0–10)	0 (0–0)	<0.001
Getting Along	25 (0–33)	0 (0–0)	0.002
Life Activities	20 (0–40)	0 (0–5)	0.001
Participation in Society	25 (17–38)	0 (0–8)	<0.001
DLQI Domains:			
Symptoms & Feelings	3 (2–4)	0 (0–0)	<0.001
Daily Activities	1 (0–2)	0 (0–0)	<0.001
Leisure	1(1–3)	0 (0–0)	<0.001
Work & School	0(0–1)	0 (0–0)	0.001
Personal Relationships	0(0–1)	0 (0–0)	0.001
Treatment	0(0–1)	0 (0–0)	0.001
LFSQQ Domains:			
Mobility	67 (58–80)	100 (88–100)	<0.001
Self-Care	100 (82–100)	100 (100–100)	0.007
Usual Activities	92 (75–100)	100 (100–100)	<0.001
Disease Burden	63 (60–75)	100 (100–100)	<0.001
Pain/Discomfort	90 (83–96)	100 (98–100)	<0.001
Psychological Health	75 (64–86)	100 (95–100)	<0.001
Social Participation	67 (85–50)	100 (100–100)	<0.001

Median value is given and 25^th^–75^th^ quartiles in parentheses.

Although the WHODAS 2.0, DLQI, and LFSQQ discriminated well between LF subjects and the control group, no global tool score was able to discriminate between stage II and stage III lymphedema subjects. LF stage discrimination was only noted with the DLQI symptoms subscale (p = 0.045) and the LFSQQ disease burden subscale (p = 0.040).

### Construct Validity

Results of the correlation analysis between the WHODAS 2.0 and the DLQI are presented in Table 7. Correlations between the WHODAS 2.0 and DLQI were generally low or moderate, including correlations between corresponding domains such as the WHODAS 2.0 life activities domain and the DLQI daily activities domain. The strongest correlation between the WHODAS 2.0 and DLQI was noted between the two total scores (r = 0.748, p<0.001).

**Table 7:** Spearman correlation coefficients among the domains of WHODAS 2.0 and DLQI.

	DLQI						
	Symptoms & Feelings	Daily Activities	Leisure	Work & School	Personal Relationships	Treatment	Total
**WHODAS 2.0**							
Cognition	0.493	0.443	0.465	0.264	0.221*	0.110*	0.516
Mobility	0.632	0.367	0.563	0.369	0.428	0.313	0.654
Self-Care	0.445	0.332	0.345	−0.061	0.298	0.259	0.405
Getting Along	0.356	0.239	0.365	−0.060	0.298	0.021*	0.327
Life Activities	0.386	0.242	0.419	0.291	0.413	0.091*	0.434
Participation	0.661	0.501	0.672	0.266	0.400	0.351	**0.708**
Total	**0.700**	0.504	**0.697**	0.389	0.454	0.259	**0.748**

* Not significantly different from zero.

Strong correlations are bolded.

The majority of correlations between WHODAS 2.0 and LFSQQ were moderate to strong (Table 8). Strong correlations were observed between the WHODAS 2.0 mobility and participation domains and LFSQQ domains. The total scores of both tools exhibited an especially high correlation (r = −0.912, p<0.001). Mobility and participation subscales of both domains displayed strong correlation with their corresponding counterparts (r = −0.917, p<0.001 and r = −0.829, p<0.001, respectively). In addition to the social participation domain of the LFSQQ, the WHODAS 2.0 participation domain also strongly correlated with the psychological health dimension of the LFSQQ (r = −0.855, p<0.001).

**Table 8:** Spearman correlation coefficients among the domains of WHODAS 2.0 and LFSQQ.

	LFSQQ							
	Mobility	Self-Care	Usual Activities	Disease Burden	Pain/Discomfort	Psychological Health	Social Participation	Total
**WHODAS 2.0**								
Cognition	−0.471	−0.391	−0.406	−0.440	−0.366	−0.671	−0.497	−0.580
Mobility	**−0.917**	−0.513	−0.518	**−0.697**	**−0.734**	−0.620	**−0.720**	**−0.860**
Self-Care	−0.513	−0.552	−0.371	−0.526	−0.427	−0.447	−0.534	−0.580
Getting Along	−0.375	−0.434	−0.271	−0.340	−0.283	−0.452	−0.501	−0.458
Life Activities	−0.521	−0.555	−0.648	−0.446	−0.411	−0.493	−0.559	−0.595
Participation	**−0.711**	−0.433	−0.632	−0.658	−0.558	**−0.855**	**−0.829**	**−0.828**
Total	**−0.821**	−0.591	**−0.696**	**−0.717**	−0.656	**−0.840**	**−0.854**	**−0.912**

Strong correlations are bolded.

Correlations between the LFSQQ and the DLQI are shown in Table 9. Total LFSQQ score highly correlated with DLQI total score (r = −0.808, p<0.001). Additionally, LFSQQ subscales related to disease burden, psychological health, and social participation strongly correlated with DLQI total score and two DLQI domains (symptoms & feelings and leisure).

**Table 9:** Spearman correlation coefficients among the domains of LFSQQ and DLQI.

	DLQI						
	Symptoms & Feelings	Daily Activities	Leisure	Work & School	Personal Relationships	Treatment	Total
**LFSQQ**							
Mobility	−0.634	−0.356	−0.562	−0.340	−0.410	−0.304	−0.648
Self-Care	−0.323	−0.320	−0.381	−0.163*	−0.248	−0.005*	−0.334
Usual Activities	−0.418	−0.436	−0.569	−0.318	−0.288	−0.007*	−0.492
Disease Burden	**−0.757**	−0.514	**−0.699**	−0.381	−0.400	−0.285	**−0.799**
Pain/Discomfort	−0.611	−0.379	−0.632	−0.307	−0.244	−0.232*	−0.648
Psychological Health	**−0.767**	−0.546	**−0.702**	−0.386	−0.363	−0.370	**−0.801**
Social Participation	**−0.730**	−0.496	**−0.692**	−0.354	−0.364	−0.319	**−0.765**
Total	**−0.778**	−0.524	**−0.731**	−0.394	−0.393	−0.321	**−0.808**

* Not significantly different from zero.

Strong correlations are bolded.

## Discussion

Comparisons of the psychometric properties of health status measures are useful in determining the appropriateness of a certain tool in a specific population. In this study, we examined the performances of three HRQoL tools (WHODAS 2.0, DLQI, LFSQQ) in the LF population and an age and gender matched control group. Although such comparisons have been performed in other chronic conditions [28]–[31], to our knowledge, our study is the first of its kind in the LF community. As such, it should be regarded as a preliminary step in bettering our understanding of the specific benefits and disadvantages of these three tools in LF research.

For all tools, feasibility was defined by the percentage of missing values per item. As the order of questionnaire administration was randomized, an ordering effect does not explain the LFSQQ's relatively low observed feasibility as compared to the other tools. Instead, the relatively high number of missing values may reflect the irrelevance of certain items to subjects. For example, the usual activities domain of the LFSQQ yielded the most missing values. This domain contained items assessing the effects of disease on activities such as agrarian work, gardening, and cleaning the floors. Because the tool does not allow for subjects to choose a “not applicable” option, subjects may have skipped items unrelated to their lifestyle rather than choose from the Likert scale ranging from “no problem” to “severe”. Questionnaire length does not seem to have an appreciable effect on the number of missing values as the 10-item DLQI performed similarly to the 36–item WHODAS 2.0. Internal consistency was acceptable (α>0.70) for all domains examined, except the LFSQQ pain/discomfort subscale (α = 0.69). Internal consistency values were highest among WHODAS 2.0 domains, and values were similar to those obtained by the WHO in a global population (α range: 0.84–0.98) [25].

Discriminant ability analysis demonstrated that the LFSQQ scales distinguished best between LF subjects and the control group. The DLQI and WHODAS 2.0 also performed well, and among all tools, the largest differences in mean scores were noted in domains relating to mobility, symptoms, and participation. Although mobility and symptoms may be directly influenced by the physical manifestations of the disease, participation in society may be an indirect effect of the disease. This finding is in agreement with other studies that demonstrate that LF affects quality of life in a more nuanced manner than solely through the disease's physical signs. Kumari et al. found that in addition to the physical burdens of LF, subjects also coped with shame and depression [4]. The psychological effects of social stigma result in delayed treatment as patients are embarrassed to reveal their condition in society [32]. These components of health status are critical to include in HRQoL measures of intervention efficacy because although a patient's physical symptoms may regress with treatment, the psychological stress and disease stigma may persist and can influence HRQoL. Current disability and HRQoL tools do not encompass all psychosocial disabilities that affect the LF patient, and including domains outside the realm of social participation in HRQoL instruments may further highlight the difficulties faced by patients in this component of HRQoL [21].

Despite the ability of the DLQI, WHODAS 2.0, and LFSQQ to discriminate between LF subjects and the control group, no global tool score was able to differentiate between stage II and stage III lymphedema. Of the domains, only the LFSQQ disease burden domain and the DLQI symptoms domain showed significant, albeit weak, association with LF stage. This weak association is most likely because the International Society of Lymphology's consensus staging system is based on the clinical assessment of disease signs rather than disability. Despite the importance of stage discrimination in the use of HRQoL as an outcome measure of intervention efficacy, the abilities of the DLQI, WHODAS 2.0, and LFSQQ to discriminate between LF stages is unknown. Conflicting results exist concerning the ability of the DLQI to distinguish between LF stages. Yahathugoda et al. noted a correlation between DLQI score and lymphedema stage, and a modified version of the DLQI was also found to correlate with LF stage [20], [21]. In addition, McPherson et al. reported decreased DLQI score following a clinical intervention, but baseline DLQI was only weakly associated with lymphedema grade [11]. Similar to our results, a study conducted in eastern India did not find a correlation between LF grade and DLQI total score [19]. The LFSQQ and WHODAS 2.0 have not been studied extensively in regard to their use in discriminating LF stage. Given our findings, the questionnaires in their current form are not sensitive to LF stage and modifications to each of the tools to better correlate with disease stage would improve each instrument's ability to accurately assess health status in LF subjects.

Correlations between global scores of all instruments were high, suggesting that the tools cover similar measures of health status. Domains of the LFSQQ and WHODAS 2.0 were highly correlated. The mobility and participation domains of the WHODAS 2.0 were differentiated by the LFSQQ. As expected, domains of different instruments covering similar content often significantly correlated. However, frequently domains assessing different facets of HRQoL correlated strongly and at times, were more highly correlated than domains of similar names. For example, the life activities domain of the WHODAS 2.0 correlated more strongly to the symptoms & feelings domain of the DLQI than the DLQI's daily activities domain. This finding may be related to the depth and breadth of topics covered by each domain. The life activities domain of the WHODAS 2.0 encompasses the effect of disease on housework and work/school in depth. The DLQI covers a broader range of topics, including the effects of disease on shopping, looking after the home, gardening, and clothing choices. This difference in extent of domain coverage may have implications on the comprehensiveness of any tool's measure of HRQoL.

As a cross-sectional study, there was no assessment of test-retest reliability of the instruments, a study limitation given the growing interest in LF treatment modalities and the increasing reliance on HRQoL tools that accurately measure change over time. In evaluating HRQoL, variations in tool applicability are expected based on differences in culture and lifestyle of targeted populations. In fact, Zeldenryk et al. noted 43 new HRQoL-related constructs in Bangladeshi focus groups that had not yet been described in the literature [33]. This study was conducted in a rural region of southern India, and thus, our results may not be generalizable to the global LF population. Finally, the study LF population was composed of only stage II and stage III lymphedema subjects. In the study region, stage I lymphedema subjects do not often present to clinic because of their minimal health changes and associated disabilities. Including stage I subjects in future studies of HRQoL tool comparison may shed light on correlations between LF stage and HRQoL.

Although many tools have been used to assess HRQoL in the LF population, no consensus has been made on the gold-standard tool. Our aim in this study was to delineate the strengths and weaknesses of the WHODAS 2.0, the DLQI, and the LFSQQ. All three tools showed moderately good performance as measured by feasibility, internal consistency, construct validity and discriminant validity. Specifically, the LFSQQ demonstrated the highest overall internal consistency, construct validity, and discriminant validity. Based on our results, the LFSQQ may be the best tool of the three to accurately assess HRQoL in the LF population. However, use of the instrument in its current state is limited by its high missing value rate. We recommend the addition of a “not applicable” option on the LFSQQ to increase tool feasibility. HRQoL largely depends on the cultural behaviors of the population studied. Before the LFSQQ can be used globally, the tool may require further modifications to take into account culturally-relevant lifestyle activities of LF-endemic areas. We recommend validation of the tool in LF populations outside of India to ensure proper application of the instrument.

Although our results suggest that the LFSQQ should be used, further research examining additional psychometric properties, respondent burden, ease of comprehension, and differences in clinically-relevant and research-relevant tools is needed to better select a HRQoL for clinical or research use.

## Supporting Information

Table S1 **Complete study dataset.** (First tab) Dataset of demographics and disease characteristics. (Second tab) Dataset of DLQI domain scores. (Third tab) Dataset of LFSQQ domain scores. (Fourth tab) Dataset of WHODAS 2.0 scores.
(XLSX)

Text S1 **Lymphatic Filariasis Quality of Life Questionnaire.**
(PDF)
