# Supplementary material for: Comparison of Three Quality of Life Instruments in Lymphatic Filariasis: DLQI, WHODAS 2.0, and LFSQQ
Source: PLoS Negl Trop Dis. 2014 Feb 20;8(2):e2716. doi: 10.1371/journal.pntd.0002716 (PMC3930502; doi:10.1371/journal.pntd.0002716)
Supplement: Text S1 — Lymphatic Filariasis Quality of Life Questionnaire. (PDF) [file pntd.0002716.s002.pdf]

LYMPHATIC FILARIASIS SPECIFIC QUALITY OF LIFE QUESTIONNAIRE (LFSQQ)

Instructions: By placing a tick in each group below, please indicate which statement best describes patient's health state in last 30 days (Do not tick more than one box in each group)

How do you rate your overall health in the past 30 Days?

**MOBILITY**					
**Domains of Indian life style**	No problem	Mild	Moderate	Severe	Most severe
Sitting & getting out of a chair					
Sitting down & standing up from floor					
Standing a long time					
Lying down over the cot					
Climbing the steps					
Putting on foot wear					
Walking a long distance					
Traveling by Indian public transport					

**SELF CARE**					
**Domains of Indian life style**	No problem	Mild	Moderate	Severe	Most severe
Dressing yourself					
Bathing					
Washing clothes yourself					
Difficulty in Using Indian toilet					
Difficulty in Using European toilet					

**USUAL ACTIVITIES**					
**Domains of Indian life style**	No problem	Mild	Moderate	Severe	Most severe
Problems in cooking					
Difficulty in cleaning the floors					
Difficulty in gardening					
Difficulty in doing agrarian work					
Difficulty in continuing the normal leisure activities					
Difficulty in continuing the job					
Difficulty in continuing the education.					

**DISEASE BURDEN**					
**Domains of Indian life style**	No problem	Mild	Moderate	Severe	Most severe
History of painful redness, swelling and cellulitis of leg (filarial fever)					
Foul smell (odour)					
Itching (Eczema/Discharge from limb)					
Wound (non-healing ulcer)					
Weight/size of the limb					
**PAIN / DISCOMFORT**					
**Domains of Indian life style**	No problem	Mild	Moderate	Severe	Most severe
Pain increases at night					
Pain increases during the day					
Pain increases while walking					
Pain increases while sitting					
Pain in the joints					
Pain in the unaffected part of the body					
Painful ulcer					

**PSYCHOLOGICAL HEALTH**					
**Domains of Indian life style**	No problem	Mild	Moderate	Severe	Most severe
Do you have sense of failure					

Fear something bad might happen to you					
Discouraged/tensed about the future					
Feeling neglected by friends and family members					
Feeling lonely					
Feeling tensed about your disease					
Difficulty in concentration & memory					

**SOCIAL PARTICIPATION**					
**Domains of Indian life style**	No problem	Mild	Moderate	Severe	Most severe
Has your life style changed after getting the disease					
Do you have problem in moving around freely without feeling self-conscious					
Do you have problem in approaching people in the community					
Do you have problem joining in social activities/get together					
How much problem you have faced because of ill health					

Instructions for Scoring and analysis:

The scoring of each question is as follows:

No problem	:	4
Mild	:	3
Moderate	:	2
Severe	:	1
Most severe	:	0
Question unanswered, not relevant	:	0

The overall QoL response is

Overall QoL = Total score/ (4 x number of questions answered) x 100

The domain score is

Domain score = Total score on domain/ (4 x number of questions answered in the domain) x 100

Note: The higher score indicates better quality of life. All questions are not relevant to every patient. So percentage analysis done based on the questions answered in each domain.

© 2009 Institute of Applied Dermatology, Kasaragod, Kerala, India www.iad.org.in
